# Effectiveness of multiple eHealth-delivered lifestyle strategies for preventing or intervening overweight/obesity among children and adolescents: A systematic review and meta-analysis

**DOI:** 10.3389/fendo.2022.999702

**Published:** 2022-09-05

**Authors:** Li-Ting Qiu, Gui-Xiang Sun, Ling Li, Ji-Dong Zhang, Dan Wang, Bo-Yan Fan

**Affiliations:** ^1^The College of Traditional Chinese Medicine, Hunan University of Chinese Medicine, Changsha, China; ^2^Provincial Key Laboratory of Traditional Chinese Medicine (TCM) Diagnostics, Hunan University of Chinese Medicine, Changsha, China; ^3^Institute of Chinese Medicine Diagnosis, Hunan University of Chinese Medicine, Changsha, China; ^4^School of Physical Education and Health, Hunan University of Technology and Business, Changsha, China

**Keywords:** eHealth, lifestyle interventions, overweight and obesity, children and adolescents, meta-analysis

## Abstract

**Objective:**

To investigate the effect of multiple eHealth-delivered lifestyle interventions on obesity-related anthropometric outcomes in children and adolescents.

**Methods:**

The Medline (*via* PubMed), Embase, Cochrane Library, Web of Science, CBM, VIP, CNKI, and Wanfang electronic databases were systematically searched from their inception to March 18, 2022, for randomized controlled trials (RCTs). Meta-analyses were performed to investigate the effect of multiple eHealth-delivered lifestyle interventions on obesity-related anthropometric outcomes (body mass index [BMI], BMI Z-score, waist circumference, body weight, and body fat%). Two independent investigators reviewed the studies for accuracy and completeness. All included studies were evaluated using the Cochrane Risk-of-Bias (ROB) Tool.

**Results:**

Forty trials comprising 6,403 patients were selected for the meta-analysis. The eligible trials were published from 2006 to 2022. Compared with the control group, the eHealth-intervention group was more effective in reducing BMI (weighted mean difference [WMD] = −0.32, 95% confidence interval [CI]: −0.50 to −0.13, *I^2^* = 85.9%), BMI Z-score (WMD = −0.08, 95% CI: −0.14 to −0.03, *I^2^* = 89.1%), waist circumference (WMD = −0.87, 95% CI: −1.70 to −0.04, *I^2^* = 43.3%), body weight (WMD = −0.96, 95% CI: −1.55 to −0.37, *I^2^* = 0.0%), and body fat% (WMD = −0.59, 95% CI: −1.08 to −0.10, *I^2^* = 0.0%). The subgroup analysis showed that parental or school involvement (WMD = −0.66, 95% CI: −0.98 to −0.34), eHealth-intervention duration of >12 weeks (WMD = −0.67, 95% CI: −0.96 to −0.38), and mobile-based interventions (WMD = −0.78, 95% CI: −1.13 to −0.43) had a significantly greater intervention effect size on BMI.

**Conclusions:**

This review recommends that multiple eHealth-delivered lifestyle strategies may be useful for preventing or treating overweight and obesity among children and adolescents. However, our results should be cautiously interpreted due to certain limitations in our study.

## Introduction

Obesity in children and adolescents has become one of the most serious public health problems of the 21^st^ century. A population-based survey from 1975 to 2016 revealed that the number of children and adolescents with obesity has rapidly increased from 11 million to 124 million, with an additional 213 million in the overweight category (1). Obesity in children and adolescents increases the incidence of chronic diseases, such as cardiovascular disease, type 2 diabetes mellitus, and cancer (2, 3). A meta-analysis suggested that obesity in children and adolescents contributes to psychological problems (4). Furthermore, children and adolescents with overweight or obesity often have a higher risk of becoming adults with obesity than those with normal weight (5), and those with a higher body mass index (BMI) have a significantly higher risk of developing a wide range of diseases in adulthood (e.g., cardiomyopathy and cancers) than those with a lower BMI (3, 6). Moreover, such conditions will increase lifetime direct healthcare and indirect productivity costs, posing a substantial financial burden worldwide (7). In this situation, implementing an effective intervention to prevent and reduce overweight/obesity among children and adolescents becomes even more important.

At present, lifestyle interventions are the mainstay for the prevention and treatment of obesity among children and adolescents. In this article, we discuss lifestyle interventions, including dietary changes (nutritional education and provision of balanced meals), physical activity (exercise promotion and reduced sedentary behaviors), behavioral therapy (cognitive behavioral therapy), or any combination of these interventions; evidence shows that single and multiple lifestyle interventions are effective in weight loss (8). However, traditional lifestyle intervention methods (e.g., hospital-based weight management (9) may not be suitable for everyone, especially those with limited time, money, or mobility. With the development of electronic, information, and communication technologies, the internet and smart devices (e.g., smartphones and tablets) provide an alternative means to engage in healthy lifestyles, thereby overcoming time, funding, and geographical barriers. Digitally delivered interventions, commonly referred to as eHealth—the use of electronic tools in delivering healthcare (10)—provide a practical and reliable method to access health information and assess, prevent, and manage health conditions. eHealth technologies, such as web-based services and mobile phone applications, can stimulate a healthy lifestyle among individuals through self-monitoring, goal setting, evaluation, and feedback or recommendation generation (11). Studies have shown that the use of internet is almost universal among teenagers (12), and it has become a major resource for the repertory of health information (13). To date, electronic-delivered health interventions are increasingly being developed and evaluated. Digital health interventions for children and adolescents have indicated significant improvements in health behaviors and self-efficacy (14); thus, eHealth technologies are a feasible channel for providing health information.

Several review studies have attempted to evaluate the effect of eHealth interventions in preventing or treating overweight and obesity among children and adolescents (15–19); however, these studies have reported inconsistent evidence and several limitations that should be considered. First, these reviews were generally limited to only one form of eHealth technology, such as web-based (15), mobile-based (16), or digital game-based interventions (17); thus, the question of whether multiple eHealth interventions (i.e., an eHealth intervention delivered in any modality focused on any particular behavior) can be considered to improve overweight and obesity in children and adolescents remains unanswered. However, only two meta-analyses have evaluated eHealth interventions involving the weight control of children and adolescents; one of these reviews (18) was limited to a few selected trials (n = 8). As the results of that review were based on limited evidence, it may not be a suitable reference, and the inclusion criteria only focused on parent-focused interventions; parents are an agent of change in improving overweight and obesity in children and adolescents. Thus, it is difficult to draw robust conclusions. Another review (19) of the inclusion criteria only included trials published in English, and the intervention duration was limited to a minimum of 6 months, during which some relevant key trials may have been missed, possibly resulting in bias. Moreover, these reviews typically focused only on a single intervention [self-monitoring (15) and physical activity promotion (16, 17)]; therefore, there is insufficient evidence on the efficacy of multiple interventions. Nevertheless, as more studies are published, the literature should be further updated. Therefore, this review aims to determine the efficacy of multiple eHealth-delivered lifestyle interventions for the prevention or treatment of overweight and obesity in children and adolescents.

## Methods

This review was developed according to the Preferred Reporting Items for Systematic Reviews and Meta-Analyses (PRISMA 2020 updated version) guidelines (20) and the Cochrane Collaboration Handbook recommendations (21). Ethical approval or patient consent was not required as all analyses were performed using the previously published studies.

We performed a comprehensive literature search in several databases, such as Medline (*via* PubMed), Embase, Cochrane Library, Web of Science, Chinese Biomedical Literature Database, Chinese Scientific Journal Database, Wanfang Data, and Chinese National Knowledge Infrastructure, to obtain all potentially eligible articles on multiple eHealth-delivered lifestyle interventions in children and adolescents with overweight or obesity from their inception to March 18, 2022. Moreover, searches were not restricted to the language or publication time.

Our search strategy was based on the Boolean logical operators by combining the Medical Subject Headings (MeSH) terms and free text-word terms. We used the following search terms: “Obesity,” “Overweight,” “Pediatric obesity,” “Telemedicine,” “eHealth,” “Children,” “Adolescents,” and “Randomized controlled trial.” Furthermore, we screened the top international journals (e.g., *Nature Reviews Endocrinology*, *Lancet Diabetes & Endocrinology*, and *JAMA Pediatrics*), famous publishers, major international conference proceedings, and gray literature (e.g., published noncommercial bibliography of doctors and masters as well as government reports) to reduce the unexpected omission of suitable lost studies that met our inclusion criterion. Reference lists of retrieved studies including these systematic reviews and meta-analyses were hand-searched to identify whether other relevant publications would meet our selection criteria. The exhaustive search strategies for each database are described in **Supplement Materials**.

Two independent investigators reviewed the studies for accuracy and completeness. The citation manager EndNote X9 (Thomson ISI Research Soft, Philadelphia, Pennsylvania, USA) was used to evaluate and filter all records. Following the assessment of titles and abstracts, the researchers obtained and reviewed the full texts of all articles. Any discrepancies between the two authors were addressed *via* discussion or consultation with a third author.

### Eligibility criteria and selection process

Inclusion criteria were defined in terms of participants, interventions, comparisons, outcomes, and study design (PICOS) criteria reporting structure as follows:

### Participants

Participants in this review were primarily children and adolescents aged 6–18 years with different body weights (underweight, healthy weight, overweight, and obese), BMI, and body composition.

### Interventions

Acceptable treatments must involve the use of eHealth (e.g., internet, computers, tablets, telehealth, mobile applications, phone calls, text messages, and emails) for delivering lifestyle interventions (e.g., dietary changes, physical activity, or behavioral therapy for weight management, such as self-monitoring, goal setting, or providing feedback), but these interventions did not have to be solely delivered through eHealth.

### Comparison

Studies were included if the control groups were treated according to standard or usual care, without any intervention, wait-list intervention, or another delivery mode (e.g., face-to-face), whereas studies were excluded if the control group used an eHealth intervention.

### Outcomes

The obesity-related anthropometric outcomes were determined (e.g., BMI, BMI Z-score, waist circumference, body weight, and body fat%).

### Study design

Only two-arm randomized controlled trials were included.

After removing the duplicates from our search, two authors independently screened titles and abstracts of the studies, and then the same two authors independently screened full manuscripts to finalize eligibility. Disagreements were resolved by discussion between the authors.

### Data collection and quality assessment

Following the Cochrane Consumers and Communication Review Group’s data extraction template guideline (21), two reviewers independently verified studies for data extraction. Based on the aims of the pre-elaborated study, we collected information on the following items: first name, year of publication, study region, study design, total, population characteristics (age and sex), intervention method, intervention duration, and study outcomes.

The overall quality of evidence for each included RCT was assessed using the Cochrane Collaboration Risk-of-Bias Tool (21). The ROB tool has several domains: sequence generation, allocation concealment, blinding of participants and personnel, blinding of outcome assessors, incomplete outcome data, selective outcome data, and other sources of bias. Based on the established criteria, each domain was rated as “low,” “unclear,” or “high.” Emerging inconsistencies were resolved by consensus through discussion.

### Statistical analyses

Data were analyzed according to the Cochrane Collaboration Handbook recommendations using a statistical software program (Stata, version 15.0; StataCorp, College Station, TX) (22). First, we measured heterogeneity within the meta-analysis using the *I^2^* statistic and p-value for heterogeneity (Cochran’s Q statistic). A Cochran’s Q test result with p-value of <0.1 indicated statistically significant heterogeneity. The *I^2^* values between 0% and 100% was used to measure the degree of heterogeneity, with threshold values of 0%–25%, 25%–50%, 50%–75%, and 75%–100% representing low, moderate, large, and extreme heterogeneity, respectively (23). Second, for dichotomous variables, the effect size was the odds ratio, whereas for continuous variables, the effect size was WMD, which were both reported with their 95% CI (24). Third, we visually evaluated the presence of publication bias using funnel plot and Egger test, with p < 0.05 indicating the presence of bias for funnel plot asymmetry. Finally, to further evaluate the heterogeneity and robustness of the results, additional subgroup analyses (parental or school involvement, type of eHealth intervention, and eHealth-intervention duration) were performed.

## Results

### Study selection and characteristics of included studies

**Figure 1:**
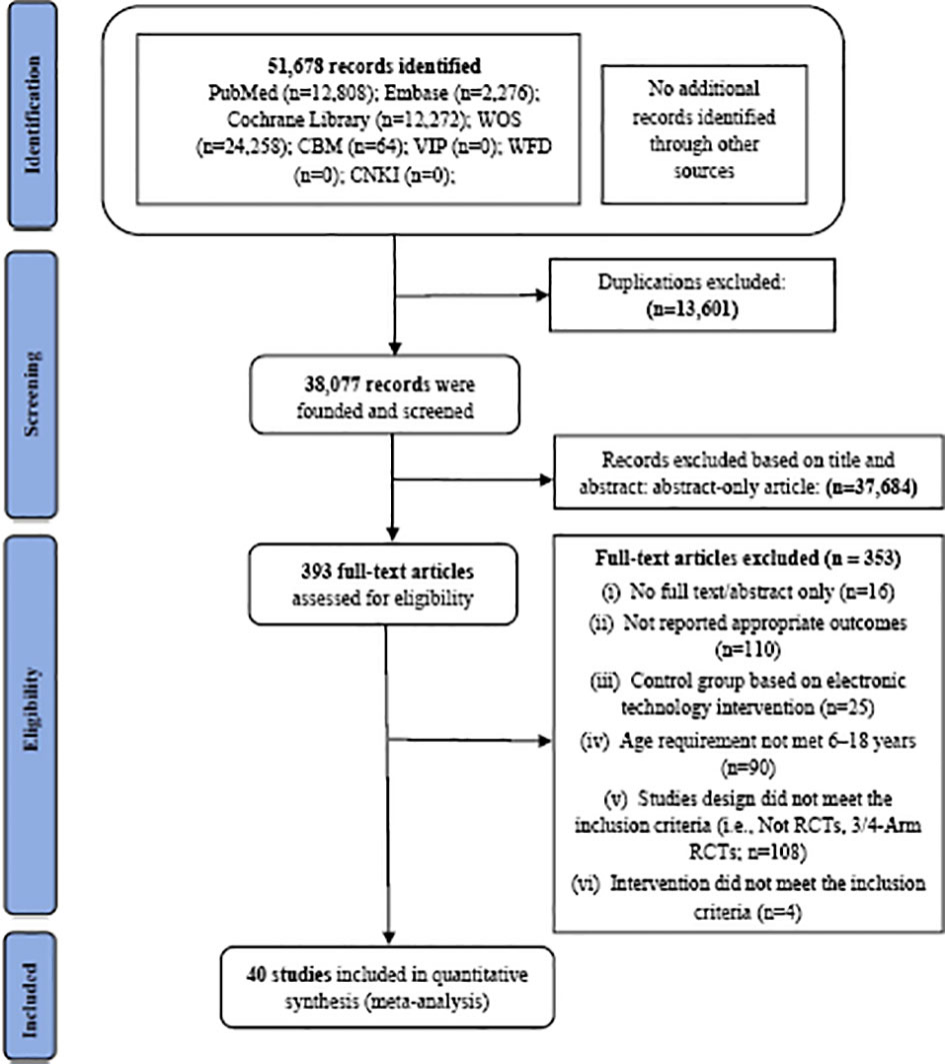
Literature review flowchart. CBM, Chinese Biomedical Literature Database; CNKI, China National Knowledge Infrastructure database; VIP, Chinese Scientific Journal Database; WFD, Wanfang database; WOS, Web of Science; RCT, randomized controlled trial.

In the initial target databases and manual search, 51,678 articles were collected. After comparing the retrieved titles, 13,601 articles were repeated and eliminated, and 38,077 remained. After screening the titles and abstracts, 393 potentially eligible articles for full-text screening were identified. Subsequently, the following articles were excluded: 16 articles with no full text or containing only the abstract, 110 studies that did not report appropriate outcomes, 25 studies that included electronic technology intervention in the control group, 90 studies where participants’ age was not within the range of 6–18 years, 108 articles with study design that did not meet the inclusion criteria (i.e., studies that were not RCTs or were 3/4-arm RCTs), and 4 studies that did not use electronic technology interventions. Finally, 40 (25–63) double-arm RCT studies were included for further meta-analysis (see **Figure 1** for details).

The eligible trials were published from 2006 to 2022. These studies were most commonly performed in the USA (16 trials). Participants were randomized to the intervention and control groups, which included 3,283 and 3,120 participants, respectively. This study comprised 3,122 males and 2,808 females (some studies did not report sex), with a mean age of 12.38 years. The duration of interventions ranged from 6 weeks to 24 months. **Table 1** presents the demographic characteristics of 40 studies.

**Table 1:** Characteristics of the studies included.

Author, Year	Country	Sample Size		Female(%)	Age M (SD)/range		eHealth-intervention Duration	Outcomes
		IG	CG		IG	CG		
Chueca et al., 2022 (25)	Spain	21	8	33.33%	10.07(0.84)		5 months	BMI; BMI Z-Score; body weight
Likhitweerawong et al. 2021(26)	Thailand	35	35	31.43%	13.11 (1.99)	12.81 (1.79)	6 months	BMI; BMI Z-Score; body weight
Bovi et al., 2021 (27)	Italy	54	49	47.57%	9.70	10.40	6 months	BMI; BMI Z-Score
Maddison et al., 2011 (28)	New Zealand	160	162	27.02%	11.60(1.10)	11.60(1.10)	6 months	BMI; BMI Z-Score; body weight; waist circumference; Body fat(%)
Graves et al., 2010 (29)	England	29	29	32.76%	9.20(0.50)	9.20(0.50)	12 weeks	BMI
Maloney et al., 2008 (30)	USA	40	20	50.00%	7.50(0.50)	7.60(0.50)	10 weeks	BMI; BMI Z-Score
Jones et al., 2008 (31)	USA	52	53	69.52%	15.00(1.00)	15.20(1.10)	16 weeks	BMI; BMI Z-Score
Doyle et al., 2008 (32)	USA	40	40	62.50%	14.90(1.70)	14.10(1.60)	16 weeks	BMI; BMI Z-Score
Heinicke et al., 2007 (33)	Australia	36	37	100.00%	14.28(1.37)	14.49(1.59)	6-8 weeks	BMI
Schiel et al., 2015(34)	Germany	34	27	52.46%	14.40(1.70)	13.00(3.10)	12 months	BMI
Rerksuppaphol et al., 2017 (70)	Thailand	111	106	51.15%	10.20(3.00)	10.00(3.10)	12 weeks	BMI Z-Score
Nollen et al., 2014 (35)	USA	26	25	100.00%	11.30(1.50)	11.30(1.70)	12 weeks	BMI
Maddison et al., 2012 (36)	New Zealand	160	162	NR	10-14		24 weeks	BMI; Body fat(%)
Likhitweerawong et al., 2020 (37)	Thailand	35	35	31.43%	13.11 (1.99)	12.81 (1.79)	2 months	BMI; body weight; waist circumference
Lau et al., 2016 (38)	China	40	40	31.25%	9.23 (0.52)		12 weeks	BMI
Chen et al., 2019 (39)	USA	23	17	42.50%	14.90 (1.67)		6 months	BMI
Baños et al., 2019 (40)	Spain	22	25	68.09%	10.43 (1.40)		10 weeks	BMI Z-Score
Staiano et al., 2018 (41)	USA	23	23	45.65%	11.20 (0.80)		24 weeks	BMI Z-Score
Staiano et al., 2017 (42)	USA	22	19	100.00%	15.30(1.20)	16.10(1.40)	12 weeks	BMI Z-Score; waist circumference
Fleischman et al., 2016 (43)	USA	21	19	77.50%	14.30 (1.90)		6 months	BMI; BMI Z-Score; body weight; waist circumference
Babic et al., 2016 (44)	Australia	167	155	65.53%	14.47(0.60)	14.33(0.50)	6 months	BMI; BMI Z-Score
Nguyen et al., 2013 (45)	Australia	73	78	NR	13-16		24 months	BMI; BMI Z-Score; body weight; waist circumference
Lubans et al., 2012 (46)	Australia	178	179	100.00%	13.15(0.44)	13.20(0.45)	12 months	BMI; BMI Z-Score; Body fat(%)
Smith et al., 2014 (47)	Australia	181	180	0.00%	12.70(0.50)	12.70(0.50)	8 months	BMI; waist circumference; Body fat(%)
Murphy et al., 2009 (48)	USA	23	12	48.57%	10.21 (1.67)		12 weeks	BMI; body weight
Wright et al., 2013 (49)	USA	24	26	42.00%	10.90(1.30)	10.50(1.20)	12 weeks	BMI; BMI Z-Score; body weight
Nawi et al., 2015 (50)	Malaysia	47	50	43.30%	16		12 weeks	BMI; waist circumference; Body fat(%)
Bagherniya et al., 2018 (51)	Iran	87	85	100.00%	13.53(0.67)	13.35(0.60)	7 months	BMI; waist circumference
Christison et al., 2016 (52)	USA	59	21	57.50%	10.10(1.30)	10.00(1.20)	6 months	BMI; BMI Z-Score; waist circumference
Coknaz et al., 2019 (53)	Turkey	53	53	56.60%	9.62(1.02)	10.31(1.15)	12 weeks	BMI; BMI Z-Score
Lubans et al., 2016 (54)	Australia	60	69	0.00%	12.70(0.50)	12.70(0.50)	8 months	BMI; BMI Z-Score; waist circumference
Currie et al., 2017 (55)	USA	34	30	56.25%	14.40 (1.92)		7 weeks	BMI Z-Score
DaSilva et al., 2019 (56)	Brazil	428	467	48.38%	14.48(1.43)	14.50(1.42)	12 months	BMI; body weight;waist circumference
Kennedy et al., 2018 (57)	Australia	353	254	50.08%	14.10(0.40)	14.20(0.50)	6 months	BMI; BMI Z-Score
Maddison et al., 2014 (58)	New Zealand	127	124	43.43%	11.20	11.30	24 weeks	BMI; BMI Z-Score; body weight; Body fat(%)
Trost et al., 2014 (59)	USA	34	41	54.67%	10.10(1.90)	9.90(1.50)	16 weeks	BMI Z-Score
Jago et al., 2006 (60)	USA	240	233	0.00%	10-14		9 weeks	BMI
Wagener et al., 2012 (61)	USA	20	20	66.70%	14.00 (1.66)		10 weeks	BMI Z-Score
Jones, 2010 (62)	USA	52	53	69.52%	15.00(1.00)	15.20(1.10)	4 months	BMI; BMI Z-Score
Li et al., 2020 (63)	China	59	59	49.15%	14.68(3.45)	14.49(3.34)	3 months	body weight

BMI, Body Mass Index; CG, control group; IG, intervention group; M, mean; NR, not Reported; SD, standard deviation.

### Quality of included studies

Seventeen of these trials (26, 28, 29, 31, 32, 37, 39, 41, 42, 44, 51, 52, 54, 55, 57–59) applied random sequence generation and allocation concealment. Based on the design, blinding of the participants and personnel in interventions was difficult to achieve in any of these studies. In 14 trials (26, 28, 31, 32, 37, 41–43, 45, 46, 48, 51, 61, 62), the outcome assessments were blinded. Finally, none of these trials showed incomplete outcome data, and 8 trials (31, 33, 34, 41, 42, 55, 57, 59) reported intention-to-treat analyses. Further details of the overall and individual quality are summarized in **Supplemental Figures S1**, **S2**.

### Meta-analyses

#### Body mass index

The meta-analysis included 32 trials evaluating the effect of intervention group and control group, and the pooled effect size estimate was based on a random-effects model, indicating that the eHealth-intervention group showed more clinical effect compared with the control group, with a statistically significant WMD of −0.32 (95% CI: −0.50 to −0.13, *I^2^* = 85.9%) (**Table 2**). The funnel plot examination showed the presence of publication bias (**Supplemental Figure S3**), and the Egger test confirmed this outcome (p < 0.05).

**Table 2:** Results of the various outcomes and subgroup analyses.

Meta-analyses variables	No. of studies	No. of patients		Pool effect size *Pooled WMDs (95% CI)*	*I*^2^
		IG	CG		
Body Mass Index	32	2,958	2,797	-0.31 (-0.50 to -0.13)	85.9%
Body Mass Index Z-Score	25	1,836	1,662	-0.08 (-0.14 to -0.03)	89.1%
Waist Circumference	11	1,173	1,185	-0.87 (-1.70 to -0.04)	43.3%
Weight	11	1,006	1,025	-0.96 (-1.55 to -0.37)	0.0%
Body Fat%	6	853	857	-0.59 (-1.08 to -0.10)	0.0%
*Subgroup analysis based on the outcome of BMI*					
*Parental or school involvement*					
overall	32	2,958	2,797	-0.31 (-0.50 to -0.13)	85.9%
Parental or school involvement, Yes	19	2,065	1,941	-0.66 (-0.98 to -0.34)	89.9%
Parental or school involvement, No	13	893	856	-0.00 (-0.22 to 0.22)	69.8%
*type of eHealth intervention*					
overall	32	2,958	2,797	-0.31 (-0.50 to -0.13)	85.9%
active video games	9	585	507	-0.04 (-0.65 to 0.57)	76.3%
Web-based	8	1,022	1,057	-0.00 (-0.07 to 0.06)	0.0%
Mobile-based	15	1,351	1,233	-0.78 (-1.13 to -0.43)	92.1%
*eHealth-intervention duration*					
overall	32	2,958	2,797	-0.31 (-0.50 to -0.13)	85.9%
> 12 weeks	21	2,365	2,237	-0.67 (-0.96 to -0.38)	89.3%
≤ 12 weeks	11	593	560	0.16 (-0.07 to 0.39)	65.7%

BMI, Body Mass Index; CI, confidence interval; CG, control group; IG, intervention group; WMD, weighted mean difference.

#### Body mass index Z-score

A total of 25 studies focused on BMI Z-score. The results of these studies showed that patients who had received eHealth interventions showed more significant changes than the control group (WMD = −0.08, 95% CI: −0.14 to −0.03, *I^2^* = 89.1%) (**Table 2**). The funnel plot did not reveal asymmetry (**Supplemental Figure S4**), and egger test suggested no publication bias (p > 0.05).

#### Waist circumference

Of the 11 studies that assessed waist circumference, pooled study results indicated that waist circumference significantly differed between the intervention and control groups (WMD = −0.87, 95% CI: −1.70 to −0.04, *I^2^* = 43.3%) (**Table 2**). The funnel plot did not identify the asymmetry (**Supplemental Figure S5**).

#### Body weight

Overall, 11 studies recorded the endpoints of body weight. High-quality evidence showed that the group with eHealth interventions was more efficient in reducing body weight than the control group (WMD = −0.96, 95% CI: −1.55 to −0.37, *I^2^* = 0.0%) (**Table 2**). The funnel plot suggested no publication bias (**Supplemental Figure S6**).

#### Body fat%

A total of six studies reported outcomes in terms of body fat%, and the body fat% was measured by bioelectrical impedance analysis (BIA) in all six studies. The results presented remarkable improvement in the intervention group compared with the control group (WMD = −0.59, 95% CI: −1.08 to −0.10, *I^2^* = 0.0%) (**Table 2**).

### Subgroup analyses

We performed several prespecified subgroup analyses (**Table 2**). A subgroup analysis revealed a significant difference in BMI in studies with parental or school involvement (WMD = −0.66, 95% CI: −0.98 to −0.34) and no statistically significant difference in trials without parental or school involvement (WMD = −0.00, 95% CI: −0.22 to 0.22). Another subgroup analysis found that the mobile-based intervention group showed a significant effect on BMI compared with the control group (WMD = −0.78, 95% CI: −1.13 to −0.43). No statistically significant differences were noted between the active video game (AVG)-based (WMD = −0.04, 95% CI: −0.65 to 0.57) and web-based (WMD = −0.00, 95% CI: −0.07 to 0.06) interventions. Further subgroup analysis demonstrated that an eHealth-intervention duration of >12 weeks (WMD = −0.67, 95% CI: −0.96 to −0.38) significantly reduced BMI, whereas that of ≤12 weeks did not have a significant effect on BMI (WMD = 0.16, 95% CI: −0.07 to 0.39).

## Discussion

This review provides the existing evidence regarding the effect of eHealth interventions through the assessment of obesity-related outcomes. Overall, the meta-analysis demonstrated the use of eHealth-delivered lifestyle interventions, which could be an effective method to improve health-related outcomes in children and adolescents and is probably more effective than the standard or usual care. The subgroup analysis indicated that parental or school involvement, eHealth-intervention duration of >12 weeks, and mobile-based interventions had significant positive intervention effects on BMI.

This meta-analysis revealed that the use of eHealth-delivered lifestyle interventions can effectively improve the BMI, BMI Z-score, waist circumference, body weight, and body fat% of children and adolescents. Our findings were consistent with those of previous systematic reviews. To date, two reviews have evaluated the effect of eHealth interventions in children/adolescents (19) and adults (64) with overweight and obesity and showed significant reductions in obesity-related outcomes, such as BMI or BMI Z-score; however, the control group in these reviews included eHealth interventions. In contrast, the control group in our review excluded any eHealth intervention. Our method may draw more meaningful and reliable conclusions to support the benefits of eHealth-delivered lifestyle intervention for children and adolescents. Lifestyle interventions through caloric restriction increased physical activity and behavior strategies, which may contribute to a negative energy balance, resulting in weight loss; this is the most popular approach to combat obesity (8). The health benefits of weight loss can reduce the risk factors for type 2 diabetes and cardiovascular diseases and improve the quality of life and mental health (65). Achieving weight control requires sustained lifestyle changes; in other words, after individuals follow a healthy lifestyle and maintain their weight loss for 2–5 years, the chances of success increase dramatically (66). However, due to the complex interaction among biological, behavioral, environmental, and cognitive factors, individuals fail to maintain weight loss over time (67). eHealth technologies provide a unique opportunity for the implementation of self-management and lifestyle modification processes for continued weight control. Based on the design of eHealth interventions, combining the persuasive system design principles and behavioral change techniques applied in eHealth-intervention design can stimulate motivation and adherence and promote healthy lifestyles and weight loss maintenance (68). Moreover, children and adolescents with overweight and obesity are desired to lose weight to improve body image, and they enjoy using technology as part of the intervention (69). A previous review demonstrated that digital health interventions improved the dietary habits, physical activity level, screen time, and psychological well-being outcomes in children and adolescents and revealed an overall significant decrease in BMI-related metrics (e.g., BMI or BMI z-score) (14). These conclusions further suggest that eHealth-delivered lifestyle interventions can enhance weight loss by promoting healthy lifestyles, which plays an important role in preventing and treating overweight and obesity. Further, a previous study reported that the eHealth intervention was effective in maintaining the relative stability of children’s weight and BMI z-score and could control the increasing prevalence of being overweight/obesity in children (70). These findings further demonstrate the important role of eHealth interventions in maintaining normal weight. However, it is worth noting that body fat% in all included studies was measured using BIA. BIA is an inexpensive, simple, and safe method to estimate body composition, which is generally considered as a portable alternative to whole-body imaging (71). However, the accuracy of BIA may easily be affected by fluid retention and general health status (71). Therefore, the results of body fat% should be interpreted with caution.

A subgroup analysis found that eHealth interventions with parental or school involvement had significantly decreased the BMI compared with those without parental or school involvement. Data from several studies indicate that parental or school involvement in obesity-related health interventions is effective in weight control among children and adolescents (72–75). Parents are usually the main influence in shaping their children’s health habits, and schools are important institutions to help children develop healthy lifestyles. The benefits of parental involvement in obesity-related eHealth interventions may be attributed to parents determining the structure of their child’s home environment (such as providing healthy foods and identifying opportunities for activity) as well as supporting and encouraging healthy behaviors (74), and the benefits of school involvement are realized in schools’ infrastructure, curriculum, policies, environment, and staff having the potential to promote health-related behaviors among children (75).Another subgroup analysis revealed that mobile-based intervention had a more significant impact on BMI than the relevant active video game (AVG) intervention and web-based intervention. The results of this analysis are differ from those of other meta-analyses (14, 17). The discrepancy in these results may be due to the study design. For instance, in our study, the participants were selected without any weight criteria, whereas a previous study only included patients with overweight or obesity (14); our study analyzed the AVG effect size on BMI, whereas a previous study assessed the BMI/zBMI data (17). Therefore, some caution is needed to interpret our results. Further subgroup analysis showed that interventions with a duration of >12 weeks significantly decreased the BMI compared with those with a duration of ≤12 weeks. This result is consistent with those of other meta-analyses (76, 77). Lifestyle intervention effects would be greater if the duration of intervention is ≥3 months (78). To prevent weight regain, a 1-year weight loss maintenance program is also recommended (79). However, it has long been recognized that children and adolescents were prone to losing interest and had poor compliance (80). Therefore, when using eHealth for long-term interventions, targeted strategies [such as sustained monitoring and goal setting, home environment support, and peer interactions (81)] should be applied to maintain long-term interest and compliance in children and adolescents; thus, children and adolescents can successfully develop healthy lifestyle habits to maintain long-term behavioral changes.

This meta-analysis has several advantages. First, our meta-analysis is the first to provide evidence for the effect of multiple eHealth-delivered lifestyle strategies in preventing and treating overweight and obesity among children and adolescents. Second, we selected an intervention tool focusing on multiple eHealth technologies (electronic communication, telephone, web, and AVGs), which provides a comprehensive outcome that determined the efficacy of the latest health technology through an approach of quantitative analysis and a reference for future research designs or practical use. Third, our review focused on comprehensive lifestyle strategies (increasing physical activity and promoting healthy eating habits, education, and guidance), providing a comprehensive overview of lifestyle interventions to prevent and treat overweight and obesity in children and adolescents. This systematic review updated the evidence from the current literature on the effect of eHealth-delivered lifestyle strategies in preventing and treating overweight and obesity among children and adolescents.

A few uncontrollable limitations must be clarified. First, it is difficult to blind the participants due to the nature of intervention; thus, the rigor of double-blind RCTs may be nonexistent in this research area, and it cannot be excluded that intervention may have an impact on the clinical evaluation. Second, although 40 individually published trials were included in this meta-analysis, some of these were obtained from the same research group (26, 28, 36, 37, 47, 54) and often used similar populations, which may directly affect the generalizability of our evidence. The third limitation is the heterogeneity ranging from mild to high, which cannot be ignored in this meta-analysis. Furthermore, we included studies with different body weights of participants (including underweight, healthy-weight, overweight, and obese) in this review, which may affect the stability of our results. Finally, a lack of evaluation and oversight have resulted in some potential quality issues related to health information available on the Internet, including inaccurate or out-of-date information, and children and adolescents may refer to inaccurate health information. To address this problem, relevant guidelines should be considered for obtaining medical information.

## Conclusion

The results of this review show a possible positive impact of eHealth-delivered lifestyle interventions on obesity-related outcomes in children and adolescents with overweight and obesity. Moreover, we found that parental or school involvement and sustained intervention over a long period significantly improved the BMI. Therefore, we cautiously suggest that practitioners, clinicians, and policymakers should consider eHealth as a model for preventing or intervening overweight and obesity in children and adolescents. More appropriate and high-quality relevant RCTs are needed in the future to determine the most effective obesity-related outcomes of eHealth interventions in preventing and treating obesity in children and adolescents.

## Author contributions

L-TQ served as principal author and had full access to all the data in the study, takes responsibility for the accuracy of the data analysis, and the integrity of the data. L-TQ, G-XS and LL contributed to the conception and design. L-TQ, J-DZ and DW contributed to data acquisition and interpretation. L-TQ, G-XS and B-YF contributed to draft of the manuscript. G-XS and LL contributed to revise of the article and final approval.

## Funding

This study was supported by the National Natural Science Foundation of China (81973670), the Natural Science Foundation of Hunan Province (2020JJ5418).

## Conflict of interest

The authors declare that the research was conducted in the absence of any commercial or financial relationships that could be construed as a potential conflict of interest.

## Publisher’s note

All claims expressed in this article are solely those of the authors and do not necessarily represent those of their affiliated organizations, or those of the publisher, the editors and the reviewers. Any product that may be evaluated in this article, or claim that may be made by its manufacturer, is not guaranteed or endorsed by the publisher.

## Supplementary material

Supplementary 1 Search strategies.

Supplementary Figure 1 Risk-of-bias graph.

Supplementary Figure 2 Risk-of-bias summary.

Supplementary Figure 3 Funnel plots of body mass index.

Supplementary Figure 4 Funnel plots of body mass index Z-score.

Supplementary Figure 5 Funnel plots of the waist circumference.

Supplementary Figure 6 Funnel plots of body weight.
