# Supplementary material for: Effectiveness of multiple eHealth-delivered lifestyle strategies for preventing or intervening overweight/obesity among children and adolescents: A systematic review and meta-analysis
Source: Front Endocrinol (Lausanne). 2022 Sep 5;13:999702. doi: 10.3389/fendo.2022.999702 (PMC9491112; doi:10.3389/fendo.2022.999702)
Supplement: Supplementary 1 — Search strategies. [file DataSheet_1.pdf]

Pubmed

#1 "Child"[Mesh]

#2 (((((((((((((Children[Title/Abstract]) OR (adolescent*[Title/Abstract])) OR (Adolescence[Title/Abstract])) OR (pediatric*[Title/Abstract])) OR (Teen*[Title/Abstract])) OR (Teenager*[Title/Abstract])) OR (Youth*[Title/Abstract])) OR (Adolescents, Female[Title/Abstract])) OR (Adolescent, Female[Title/Abstract])) OR (Female Adolescent[Title/Abstract])) OR (Female Adolescents[Title/Abstract])) OR (Adolescents, Male[Title/Abstract])) OR (Adolescent, Male[Title/Abstract])) OR (Male Adolescent[Title/Abstract])) OR (Male Adolescents[Title/Abstract])

#3 #1 OR #2

#4 "Telemedicine"[Mesh]

#5 ((Mobile Health[Title/Abstract]) OR (Health[Title/Abstract])) OR (Mobile[Title/Abstract])) OR (mHealth[Title/Abstract])) OR (Telehealth[Title/Abstract])) OR (eHealth[Title/Abstract])) OR (Electronic Health[Title/Abstract])) OR (ICT[Title/Abstract])) OR (Internet[Title/Abstract])) OR (web[Title/Abstract])) OR (online[Title/Abstract])) OR (computer[Title/Abstract])) OR (social media[Title/Abstract])) OR (social media health[Title/Abstract])) OR (mobile[Title/Abstract])) OR (smartphone[Title/Abstract])) OR (app[Title/Abstract])) OR (mobile apps[Title/Abstract])) OR (portable electronic app[Title/Abstract])) OR (portables oftware app[Title/Abstract])) OR (application[Title/Abstract])) OR (mobile applications[Title/Abstract])) OR (Digital Health[Title/Abstract])) OR (mobile technology[Title/Abstract])) OR (cellular phone[Title/Abstract])) OR (telecommunications[Title/Abstract])) OR (web-based[Title/Abstract])) OR (text message[Title/Abstract])) OR (SMS[Title/Abstract])) OR (short message service[Title/Abstract])) OR (portable game[Title/Abstract])) OR (computers, handheld[Title/Abstract])) OR (PDA[Title/Abstract])) OR (personal digital assistant[Title/Abstract])) OR (Twitter[Title/Abstract])) OR (tweets[Title/Abstract])) OR (Facebook[Title/Abstract])) OR (Instagram[Title/Abstract])) OR (mobile fitness apps[Title/Abstract])) OR (online social networking[Title/Abstract])) OR (virtual

reality[Title/Abstract])) OR (avatars[Title/Abstract])) OR (online gaming[Title/Abstract])) OR (video games[Title/Abstract])) OR (information[Title/Abstract] AND communication technology[Title/Abstract])

#6 #4 OR #5

#7 "Obesity"[Mesh]

#8 ((((adiposity[Title/Abstract]) OR (obese[Title/Abstract])) OR (weight[Title/Abstract])) OR (overweight[Title/Abstract])) OR (eutrophic individuals[Title/Abstract])

#9 #7 OR #8

#10 (randomized controlled trial [pt] OR controlled clinical trial [pt] OR randomized [tiab] OR placebo [tiab] OR drug therapy [sh] OR randomly [tiab] OR trial [tiab] OR groups [tiab]) NOT (animals [mh] NOT humans [mh])

#11 "Pediatric Obesity"[Mesh]

#13 #3 AND #6 AND #9 AND #10

#14 #6 AND #10 AND #11

Embase

#1 'child'/exp OR child

#2 'children':ab,ti OR 'adolescent*':ab,ti OR 'adolescence':ab,ti OR 'pediatric*':ab,ti OR 'teen*':ab,ti OR 'teenager*':ab,ti OR 'youth*':ab,ti OR 'adolescents, female':ab,ti OR 'adolescent, female':ab,ti OR 'female adolescent':ab,ti OR 'female adolescents':ab,ti OR 'adolescents, male':ab,ti OR 'adolescent,male':ab,ti OR 'male adolescent':ab,ti OR 'male adolescents':ab,ti

#3 #1 OR #2

#4 'telemedicine'/exp OR telemedicine

#5 'mobile health':ab,ti OR 'health':ab,ti OR 'mhealth':ab,ti OR 'telehealth':ab,ti OR 'ehealth':ab,ti OR 'electronic health':ab,ti OR 'ict':ab,ti OR 'information and communication technology':ab,ti OR 'internet':ab,ti OR 'web':ab,ti OR 'online':ab,ti

OR 'computer':ab,ti OR 'social media':ab,ti OR 'social media health':ab,ti OR 'mobile':ab,ti OR 'smartphone':ab,ti OR 'app':ab,ti OR 'mobile apps':ab,ti OR 'portable electronic app':ab,ti OR 'portables oftware app':ab,ti OR 'application':ab,ti OR 'mobile applications':ab,ti OR 'digital health':ab,ti OR 'mobile technology':ab,ti OR 'cellular phone':ab,ti OR 'telecommunications':ab,ti OR 'web-based':ab,ti OR 'text message':ab,ti OR 'sms':ab,ti OR 'short message service':ab,ti OR 'portable game':ab,ti OR 'computers,handheld':ab,ti OR 'pda':ab,ti OR 'personal digital assistant':ab,ti OR 'twitter':ab,ti OR 'tweets':ab,ti OR 'facebook':ab,ti OR 'instagram':ab,ti OR 'mobile fitness apps':ab,ti OR 'online social networking':ab,ti OR 'virtual reality':ab,ti OR 'avatars':ab,ti OR 'online gaming':ab,ti OR 'video games':ab,ti

#6 #4 OR #5

#7 'obesity'/exp OR obesity

#8 'adiposity':ab,ti OR 'obese':ab,ti OR 'overweight':ab,ti OR 'eutrophic individuals':ab,ti

#9 #7 OR #8

#10 'crossover procedure':de OR 'double-blind procedure':de OR 'randomized controlled trial':de OR 'single-blind procedure':de OR (random* OR factorial* OR crossover* OR cross NEXT/1 over* OR placebo* OR doubl* NEAR/1 blind* OR singl* NEAR/1 blind* OR assign* OR allocat* OR volunteer*):de,ab,ti

#11 ('pediatrics'/exp OR pediatrics) AND ('obesity'/exp OR obesity)

#13 #3 AND #6 AND #9 AND #10

#14 #6 AND #10 AND #11

Cochrane Library

#1 MeSH descriptor: [Child] explode all trees

#2 (Children):ti,ab,kw OR (adolescent*):ti,ab,kw OR (Adolescence):ti,ab,kw OR (pediatric*):ti,ab,kw OR (Teen*):ti,ab,kw OR (Youth*):ti,ab,kw OR (Teenager*):ti,ab,kw OR (Adolescents, Female):ti,ab,kw OR (Adolescent,

Female):ti,ab,kw OR (Female Adolescent):ti,ab,kw OR (Female Adolescents):ti,ab,kw OR (Adolescents, Male):ti,ab,kw OR (Adolescent, Male):ti,ab,kw OR (Male Adolescent):ti,ab,kw OR (Male Adolescents):ti,ab,kw

#3 #1 OR #2

#4 MeSH descriptor: [Telemedicine] explode all trees

#5 (Mobile Health):ab,ti,kw OR (Health):ab,ti,kw OR (Mobile):ab,ti,kw OR (mHealth):ab,ti,kw OR (Telehealth):ab,ti,kw OR (eHealth):ab,ti,kw OR (Electronic Health):ab,ti,kw OR (ICT):ab,ti,kw OR (information and communication technology):ab,ti,kw OR (Internet):ab,ti,kw OR (web):ab,ti,kw OR (online):ab,ti,kw OR (computer):ab,ti,kw OR (social media):ab,ti,kw OR (social media health):ab,ti,kw OR (mobile):ab,ti,kw OR (smartphone):ab,ti,kw OR (app):ab,ti,kw OR (mobile apps):ab,ti,kw OR (portable electronic app):ab,ti,kw OR (portables oftware app):ab,ti,kw OR (application):ab,ti,kw OR (mobile applications):ab,ti,kw OR (Digital Health):ab,ti,kw OR (mobile technology):ab,ti,kw OR (cellular phone):ab,ti,kw OR (telecommunications):ab,ti,kw OR (web-based):ab,ti,kw OR (text message):ab,ti,kw OR (SMS):ab,ti,kw OR (short message service):ab,ti,kw OR (portable game):ab,ti,kw OR (computers, handheld):ab,ti,kw OR (PDA):ab,ti,kw OR (personal digital assistant):ab,ti,kw OR (Twitter):ab,ti,kw OR (tweets):ab,ti,kw OR (Facebook):ab,ti,kw OR (Instagram):ab,ti,kw OR (mobile fitness apps):ab,ti,kw OR (online social networking):ab,ti,kw OR (virtual reality):ab,ti,kw OR (avatars):ab,ti,kw OR (online gaming):ab,ti,kw OR (video games):ab,ti,kw

#6 #4 OR #5

#7 MeSH descriptor: [Obesity] explode all trees

#8 (adiposity):ab,ti,kw OR (obese):ab,ti,kw OR (weight):ab,ti,kw OR (overweight):ab,ti,kw OR (eutrophic individuals):ab,ti,kw

#9 #7 OR #8

#10 MeSH descriptor: [Pediatric Obesity] explode all trees

#11 #3 AND #6 AND #9

#12 #6 AND #10

Web of science

#1 ((((((((((((((TS=(child)) OR TS=(Children)) OR TS=(adolescent*)) OR TS=(Adolescence)) OR TS=(pediatric*)) OR TS=(Teen*)) OR TS=(Teenager*)) OR TS=(Youth*)) OR TS=(Adolescents, Female)) OR TS=(Adolescent, Female)) OR TS=(Female Adolescent)) OR TS=(Female Adolescents)) OR TS=(Adolescents, Male)) OR TS=(Adolescent, Male)) OR TS=(Male Adolescent)) OR TS=(Male Adolescents)

#2 ((TS=(Telemedicine)) OR TS=(Mobile Health)) OR TS=(Health)) OR TS=(Mobile)) OR TS=(mHealth)) OR TS=(Telehealth)) OR TS=(eHealth)) OR TS=(Electronic Health)) OR TS=(ICT)) OR TS=(information and communication technology)) OR TS=(Internet)) OR TS=(web)) OR TS=(online)) OR TS=(computer)) OR TS=(social media)) OR TS=(social media health)) OR TS=(mobile)) OR TS=(smartphone)) OR TS=(app)) OR TS=(mobile apps)) OR TS=(portable electronic app)) OR TS=(portables oftware app)) OR TS=(application)) OR TS=(mobile applications)) OR TS=(Digital Health)) OR TS=(mobile technology)) OR TS=(cellular phone)) OR TS=(telecommunications)) OR TS=(web-based)) OR TS=(text message)) OR TS=(SMS)) OR TS=(short message service)) OR TS=(portable game)) OR TS=(computers, handheld)) OR TS=(PDA)) OR TS=(personal digital assistant)) OR TS=(Twitter)) OR TS=(tweets)) OR TS=(Facebook)) OR TS=(Instagram)) OR TS=(mobile fitness apps)) OR TS=(online social networking)) OR TS=(virtual reality)) OR TS=(avatars)) OR TS=(online gaming)) OR TS=(video games)

#3 ((((((((((TS=(clinical trial*)) OR TS=(research design)) OR TS=(comparative stud*)) OR TS=(evaluation stud*)) OR TS=(controlled trial*)) OR TS=(follow-up stud*)) OR TS=(prospective stud*)) OR TS=(random*)) OR TS=(placebo*)) OR TS=(single blind*)) OR TS=(double blind*)

#4 (((((TS=(obesity)) OR TS=(adiposity)) OR TS=(obese)) OR TS=(weight)) OR

TS=(overweight)) OR TS=(eutrophic individuals)

#5 TS=(pediatric obesity)

#6 #1 AND #2 AND #3 AND #4

#7 #2 AND #3 AND #5

Chinese Biomedical Literature database Search strategy

#1 "儿童"[不加权:扩展]

#2 "儿童"[常用字段:智能] OR "青少年"[常用字段:智能] OR "幼儿"[常用字段:智能] OR "小儿"[常用字段:智能] OR "未成年人"[常用字段:智能] OR "青年"[常用字段:智能]

#3 (#2) OR (#1)

#3 "远程医学"[不加权:扩展]

#4 "移动健康"[常用字段:智能] OR "互联网医疗"[常用字段:智能] OR "APP"[常用字段:智能] OR "微信"[常用字段:智能] OR "互联网"[常用字段:智能] OR "网络"[常用字段:智能] OR "因特网"[常用字段:智能] OR "计算机互联网"[常用字段:智能] OR "国际互联网"[常用字段:智能]

#5 "小程序"[常用字段:智能] OR "应用程序"[常用字段:智能] OR "移动医疗"[常用字段:智能] OR "手机"[常用字段:智能] OR "智能手机"[常用字段:智能] OR "便携式电话"[常用字段:智能] OR "移动电话"[常用字段:智能] OR "短信"[常用字段:智能] OR "信息和通信技术"[常用字段:智能]

#6 "移动设备"[常用字段:智能] OR "平台"[常用字段:智能]

#7 (#6) OR (#5) OR (#4) OR (#3)

#8 "肥胖症"[不加权:扩展]

#9 "肥胖病"[常用字段:智能] OR "肥胖"[常用字段:智能] OR "体重"[常用字段:智能] OR "超重"[常用字段:智能]

#10 (#9) OR (#8)

#11 "随机对照试验"[不加权:扩展]

#12 "随机对照实验"[常用字段:智能] OR "随机分组"[常用字段:智能] OR "随机"[常用字段:智能] OR "RCT"[常用字段:智能] OR "随机对照研究"[常用字段:智能] OR "随机对照"[常用字段:智能] OR "rct"[常用字段:智能]

#13 (#12) OR (#11)

#14 (#13) AND (#10) AND (#7) AND (#3)

Chinese Scientific Journal database Search strategy

(((((((题名或关键词=儿童 OR 题名或关键词=青少年) OR 题名或关键词=幼儿) OR 题名或关键词=小儿) OR 题名或关键词=未成年人) OR 题名或关键词=青年) AND ((((((((((((((((((题名或关键词=远程医学 OR 题名或关键词=移动健康) OR 题名或关键词=互联网医疗) OR 题名或关键词=APP) OR 题名或关键词=微信) OR 题名或关键词=互联网) OR 题名或关键词=网络) OR 题名或关键词=因特网) OR 题名或关键词=计算机互联网) OR 题名或关键词=国际互联网) OR 题名或关键词=小程序) OR 题名或关键词=应用程序) OR 题名或关键词=移动医疗) OR 题名或关键词=手机) OR 题名或关键词=智能手机) OR 题名或关键词=便携式电话) OR 题名或关键词=移动电话) OR 题名或关键词=短信) OR 题名或关键词=信息和通信技术) OR 题名或关键词=移动设备) OR 题名或关键词=平台)) AND ((((题名或关键词=肥胖症 OR 题名或关键词=肥胖病) OR 题名或关键词=肥胖) OR 题名或关键词=体重) OR 题名或关键词=超重)) AND (((((((题名或关键词=随机对照试验 OR 题名或关键词=随机对照实验) OR 题名或关键词=随机分组) OR 题名或关键词=随机) OR 题名或关键词=RCT) OR 题名或关键词=随机对照研究) OR 题名或关键词=随机对照) OR 题名或关键词=rct))

Wanfang database search strategy

主题:(儿童+青少年+幼儿+小儿+未成年人+青年) and 主题:(远程医学+移动健康

+互联网医疗+APP+微信+互联网+网络+因特网+计算机互联网+国际互联网+小程序+应用程序+移动医疗+手机+智能手机+便携式电话+移动电话+短信+信息和通信技术+移动设备+平台) and 主题:(肥胖症+肥胖病+肥胖+体重+超重) and 主题:(随机对照试验+随机对照实验+随机分组+随机+RCT+随机对照研究+随机对照+rct)

China National Knowledge Infrastructure database Search strategy

((((主题=儿童 或者 题名=儿童 或者 v_subject=中英文扩展(儿童) 或者 title=中英文扩展(儿童)) 或者 (主题=青少年 或者 题名=青少年 或者 v_subject=中英文扩展(青少年) 或者 title=中英文扩展(青少年))) 或者 ((主题=幼儿 或者 题名=幼儿 或者 v_subject=中英文扩展(幼儿) 或者 title=中英文扩展(幼儿)) 或者 (主题=小儿 或者 题名=小儿 或者 v_subject=中英文扩展(小儿) 或者 title=中英文扩展(小儿)))) 或者 ((主题=未成年人 或者 题名=未成年人 或者 v_subject=中英文扩展(未成年人) 或者 title=中英文扩展(未成年人)) 或者 (主题=青年 或者 题名=青年 或者 v_subject=中英文扩展(青年) 或者 title=中英文扩展(青年)))) 并且 (FILETYPE=DS) 并且 ((((((((主题=远程医学 或者 题名=远程医学 或者 v_subject=中英文扩展(远程医学) 或者 title=中英文扩展(远程医学)) 或者 (主题=移动健康 或者 题名=移动健康 或者 v_subject=中英文扩展(移动健康) 或者 title=中英文扩展(移动健康))) 或者 ((主题=互联网医疗 或者 题名=互联网医疗 或者 v_subject=中英文扩展(互联网医疗) 或者 title=中英文扩展(互联网医疗)) 或者 (主题=中英文扩展(APP) 或者 题名=中英文扩展(APP) 或者 v_subject=APP 或者 title=APP))) 或者 ((主题=移动医疗 或者 题名=移动医疗 或者 v_subject=中英文扩展(移动医疗) 或者 title=中英文扩展(移动医疗)) 或者 (主题=应用程序 或者 题名=应用程序 或者 v_subject=中英文扩展(应用程序) 或者 title=中英文扩展(应用程序)))) 或者 ((主题=微信 或者 题名=微信 或者 v_subject=中英文扩展(微信) 或者 title=中英文扩展(微信)) 或者

(主题=互联网 或者 题名=互联网 或者 v_subject=中英文扩展(互联网) 或者 title=中英文扩展(互联网)))) 或者 ((主题=手机 或者 题名=手机 或者 v_subject=中英文扩展(手机) 或者 title=中英文扩展(手机)) 或者 (主题=小程序 或者 题名=小程序 或者 v_subject=中英文扩展(小程序) 或者 title=中英文扩展(小程序)))) 或者 ((主题=平台 或者 题名=平台 或者 v_subject=中英文扩展(平台) 或者 title=中英文扩展(平台)) 或者 (主题=短信 或者 题名=短信 或者 v_subject=中英文扩展(短信) 或者 title=中英文扩展(短信)))) 或者 ((主题=移动电话 或者 题名=移动电话 或者 v_subject=中英文扩展(移动电话) 或者 title=中英文扩展(移动电话)) 或者 (主题=移动设备 或者 题名=移动设备 或者 v_subject=中英文扩展(移动设备) 或者 title=中英文扩展(移动设备)))) 并且 (FILETYPE=DS) 并且 ((((主题=肥胖症 或者 题名=肥胖症 或者 v_subject=中英文扩展(肥胖症) 或者 title=中英文扩展(肥胖症)) 或者 (主题=肥胖病 或者 题名=肥胖病 或者 v_subject=中英文扩展(肥胖病) 或者 title=中英文扩展(肥胖病))) 或者 ((主题=肥胖 或者 题名=肥胖 或者 v_subject=中英文扩展(肥胖) 或者 title=中英文扩展(肥胖)) 或者 (主题=体重 或者 题名=体重 或者 v_subject=中英文扩展(体重) 或者 title=中英文扩展(体重)))) 或者 (主题=超重 或者 题名=超重 或者 v_subject=中英文扩展(超重) 或者 title=中英文扩展(超重))) (模糊匹配)
