# Supplementary figures and images for: Effectiveness of multiple eHealth-delivered lifestyle strategies for preventing or intervening overweight/obesity among children and adolescents: A systematic review and meta-analysis
Source: Front Endocrinol (Lausanne). 2022 Sep 5;13:999702. doi: 10.3389/fendo.2022.999702 (PMC9491112; doi:10.3389/fendo.2022.999702)

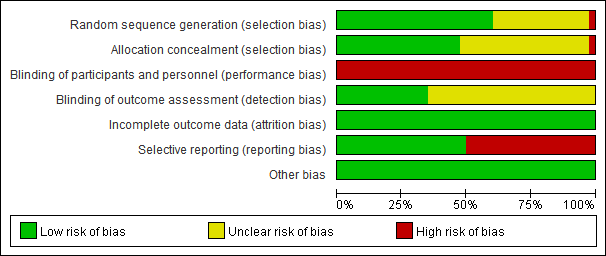

Supplement: Supplementary Figure 1 — Risk-of-bias graph. [file Image_1.tif]

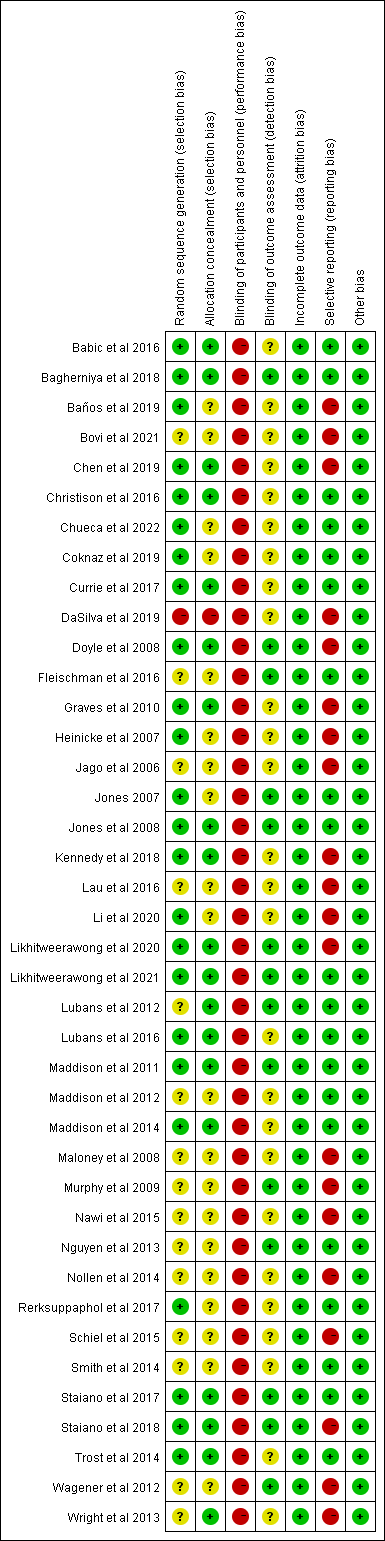

Supplement: Supplementary Figure 2 — Risk-of-bias summary. [file Image_2.tif]

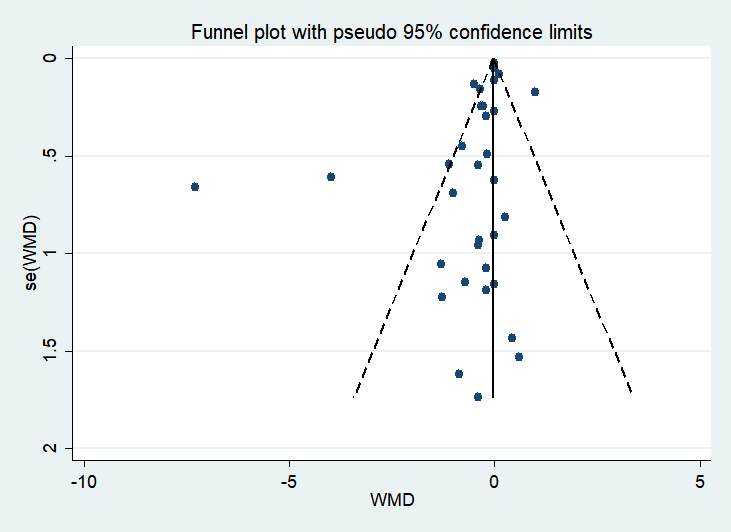

Supplement: Supplementary Figure 3 — Funnel plots of body mass index. [file Image_3.tif]

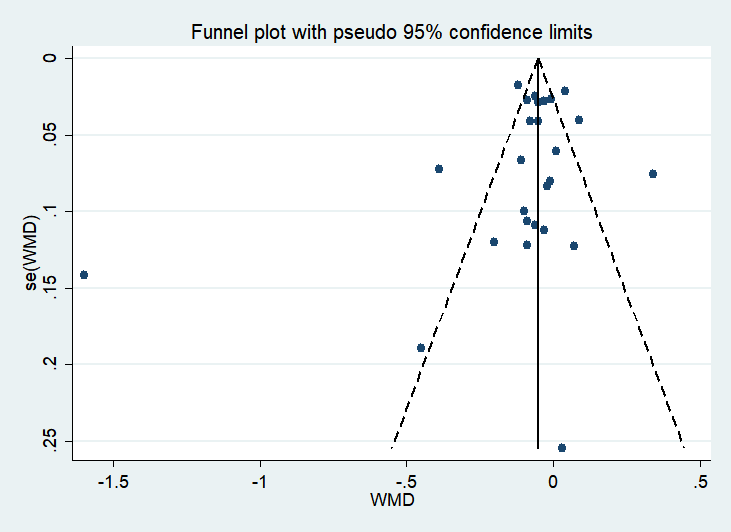

Supplement: Supplementary Figure 4 — Funnel plots of body mass index Z-score. [file Image_4.tif]

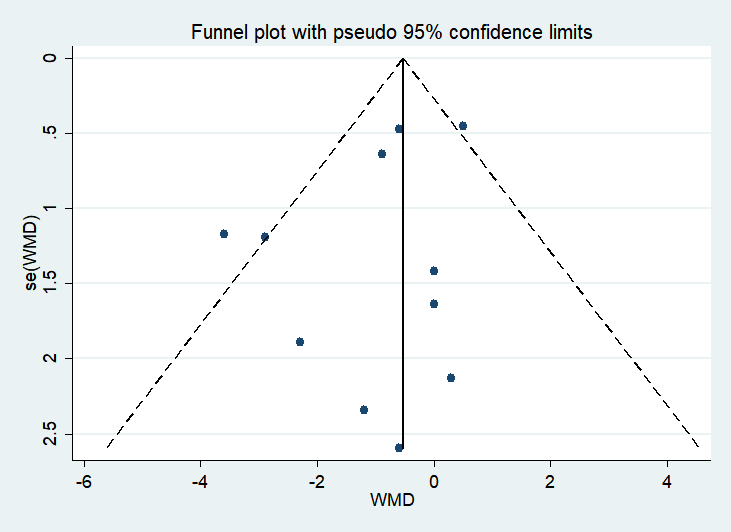

Supplement: Supplementary Figure 5 — Funnel plots of the waist circumference. [file Image_5.tif]

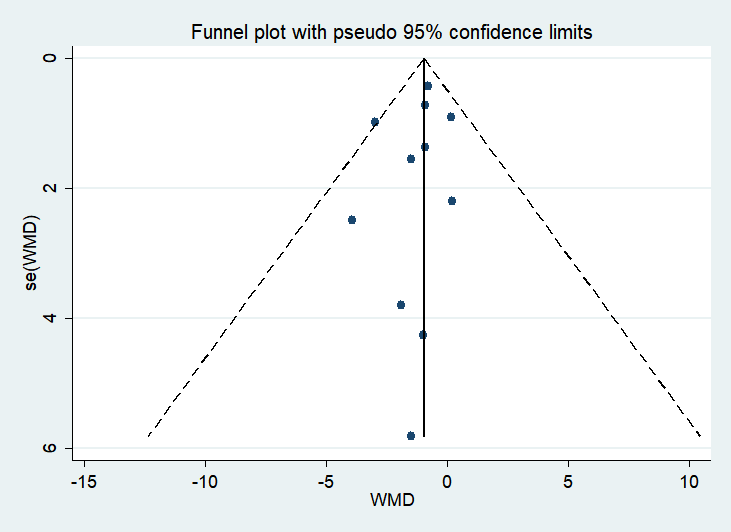

Supplement: Supplementary Figure 6 — Funnel plots of body weight. [file Image_6.tif]
